# Identification and validation of prognosis-associated DNA repair gene signatures in colorectal cancer

**DOI:** 10.1038/s41598-022-10561-w

**Published:** 2022-04-28

**Authors:** Dingli Song, Dai Zhang, Sisi Chen, Jie Wu, Qian Hao, Lili Zhao, Hong Ren, Ning Du

**Affiliations:** 1 Department of Thoracic Surgery, The First Affiliated Hospital, Xi’an Jiaotong University, Xi’an, 710061 Shaanxi China; 2 Department of Oncology, The Second Affiliated Hospital, Xi’an Jiaotong University, Xi’an, China; 3 Department of Neurology, The Second Affiliated Hospital, Xi’an Jiaotong University, Xi’an, China

**Keywords:** Cancer, Genetics, Immunology

## Abstract

Colorectal cancer (CRC) is the third most common malignant tumor. DNA damage plays a crucial role in tumorigenesis, and abnormal DNA repair pathways affect the occurrence and progression of CRC. In the current study, we aimed to construct a DNA repair-related gene (DRG) signature to predict the overall survival (OS) of patients with CRC patients. The differentially expressed DRGs (DE-DRGs) were analyzed using The Cancer Genome Atlas (TCGA) and Gene Expression Omnibus (GEO) databases. The prognostic gene signature was identified by univariate Cox regression and least absolute shrinkage and selection operator (LASSO)-penalized Cox proportional hazards regression analysis. The predictive ability of the model was evaluated using the Kaplan–Meier curves and time-dependent receiver operating characteristic (ROC) curves. The gene set enrichment analysis (GSEA) was performed to explore the underlying biological processes and signaling pathways. ESTIMATE and CIBERSORT were implemented to estimate the tumor immune score and immune cell infiltration status between the different risk group. The half-maximal inhibitory concentration (IC50) was evaluated to representing the drug response of this signature. Nine DE-DRGs (ESCO2, AXIN2, PLK1, CDC25C, IGF1, TREX2, ALKBH2, ESR1 and MC1R) signatures was constructed to classify patients into high- and low-risk groups. The risk score was an independent prognostic indicator of OS (hazard ratio > 1, P < 0.001). The genetic alteration analysis indicated that the nine DE-DRGs in the signature were changed in 63 required samples (100%), and the major alteration was missense mutation. Function enrichment analysis revealed that the immune response and mtotic sister chromatid segregation were the main biological processes. The high-risk group had higher immune score than the low-risk group. What’s more, low-risk patients were more sensitive to selumetinib and dasatinib. The nine DE-DRGs signature was significantly associated with OS and provided a new insight for the diagnosis and treatment of CRC.

## Introduction

Colorectal cancer (CRC) is the third most common malignant tumor, and the account for 10% of annual cancer-related deaths worldwide in 2018^1^.The incidence and mortality rates in men are four times higher than those in women^2^. More than 20% of patients with CRC are diagnosed at a progressive stage because of the inapparent early symptoms, leading to poor prognosis^3^. The specific mechanism of CRC is still unclear, but there are numerous risk factors identified for CRC occurrence and development, including hereditary CRC, positive family history, inflammatory bowel disease, male sex, smoking, drinking, and red meat intake, which involve complicated shifts at different molecular levels^4^. Although the diagnosis and treatment of CRC have made great progress, the precise treatment still faces a huge challenge. Therefore, it is essential to find new reliable prognostic biomarkers and molecular alterations to develop more effective treatments for CRC.

Human cells are exposed to multiple sources of DNA damage, including radical oxygen species, diet, ultraviolet radiation, and numerous drugs, resulting in various DNA damage responses (DDRs)^5^. Previous studies have identified the crucial roles played by DDR in tumorigenesis, premature aging, chronic inflammation, and apoptosis^6,7^. Specific alterations in the DNA repair pathway can also influence the occurrence and progression of CRCs^8^. To date, DDR mainly includes single-strand and double-strand repair. Numerous DNA damage repair genes, such as *MSH2, MSH6, MLH1, PMS2, CHEK2,* and *PALB2*, have an integral mechanistic effect on DNA repair pathways^9^. A recent study evaluated a prognostic model based on eight DNA repair-related genes (DRGs) to predict the prognosis of patients with breast cancer; the DRGs showed robust predictive power^10^. Another study found that *ARID1A* mutation in CRC is strongly related to the DNA repair pathway and affects the patients’ clinical outcomes^11^. Some studies have focused on the association of multiple gene signatures with the prognosis of CRC, such as immune genes and metabolism-related genes^12,13^. However, few studies have combined differential DRGs with the prognosis of CRC to construct a prognostic prediction model. It is highly important to explore the status of DRGs for predicting CRC outcomes.

In our study, we established a different signature based on DRGs to predict the overall survival (OS) of patients with CRC using TCGA colon and rectal cancer cohorts. We then we validated the DRGs signature in the GEO database (GSE87211, GSE103479). The CRC-specific prognostic model provides a novel direction for the diagnosis and treatment of CRC. In addition, we used GSEA and CIBERSORT to explore the underlying biological functions and immune cell infiltration status of DRGs in CRC, which may provide new insights into tumorigenesis and development.

## Results

### Patients features

**Figure 1:**
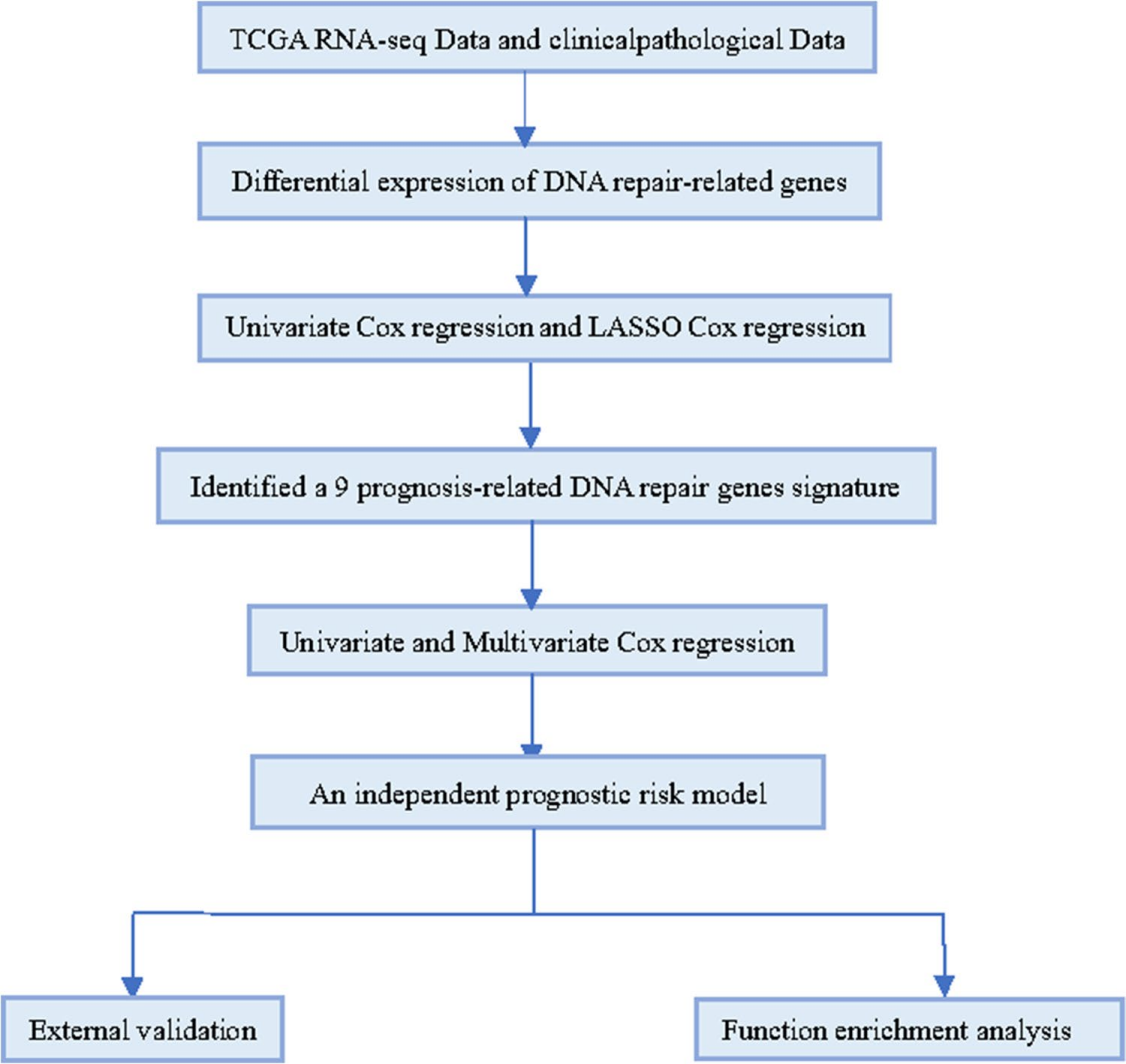
The flowchart of the research design.

The flowchart of this study design was displayed in Fig. 1.

TCGA colon and rectal cohorts contained 530 samples (488 patients with CRC and 42 normal samples) to identify the predictive signature. We excluded samples with incomplete clinical information (age, sex, survival time, survival status and TNM stage), and a total of 396 cancer patients were used to further estimate the independence of the predictive model. The GSE87211 dataset included 363 samples (203 rectal tumors and 160 rectal normal samples), and the GSE103479 dataset consisted of 177 patients with CRC. A total of 380 patients with CRC were acquired from GEO after merging the two datasets. Subsequently, 338 patients with complete clinical information were screened in this study. Detailed basic clinical information of these patients is presented in Table S1.

### Identification of prognostic DNA repair-related DEGs in patients from TCGA

**Figure 2:**
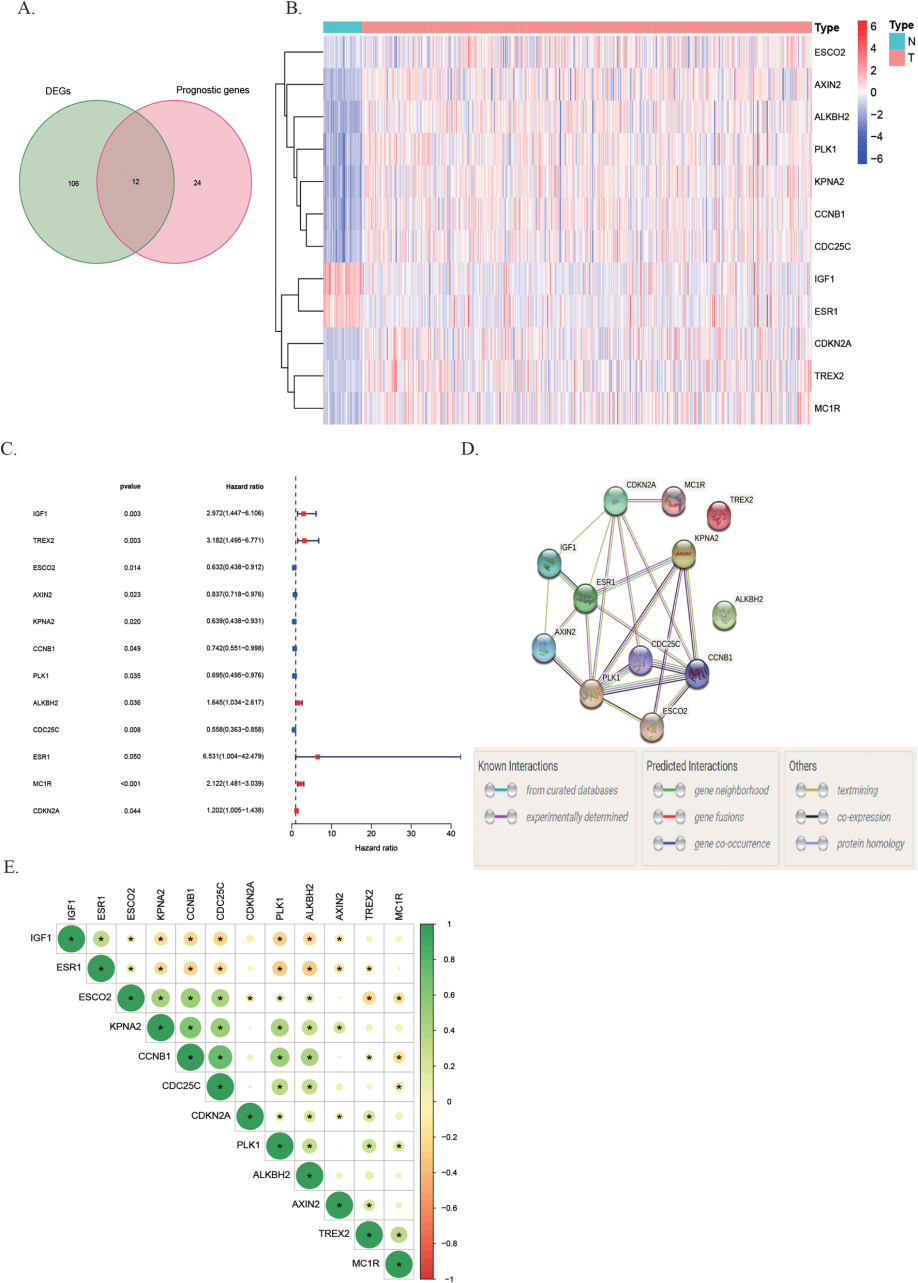
Identification of prognostic DNA repair-related DEGs in TCGA. **(A)** Venn plot to identify prognostic DE-DRGs in CRC based on data from TCGA; **(B)** the expression patterns of the 12 DE-DRGs in a heatmap; **(C)** forest plots of 12 DE-DRGs associated with OS by univariate Cox regression. **(D)** The 12 DE-DRGs interactions of PPI network downloaded from STRING database. **(E)** The correlation heatmap of 12 DE-DRGs. The different colors presented correlation coefficients.

A total of 493 of 513 DRGs were found in TCGA CRC cohort (Table S2). According to the screening criteria, 118 DE-DRGs containing 9 downregulated and 109 upregulated genes were identified between 488 CRC and 42 non-cancer tissues using the Wilcoxon signed-rank test (Fig. S1, Table S3). About 36 DRGs were related to survival in the univariate Cox regression analysis (*P* < 0.05, Fig. S2). Finally, 12 DE-DRGs associated with OS were presented in this study (Fig. 2A–C). The protein–protein interaction network among the 12 genes was draw through STRING and we used cytoHubba to identify that *ESR1, PLK1, CDKN2A* and *CCNB1* were the hub genes (interaction score: 0.40, Fig. 2D). The correlation strength between these genes is shown in Fig. 2E.

### Construction of a DRGs prognostic signature in the TCGA cohort

**Figure 3:**
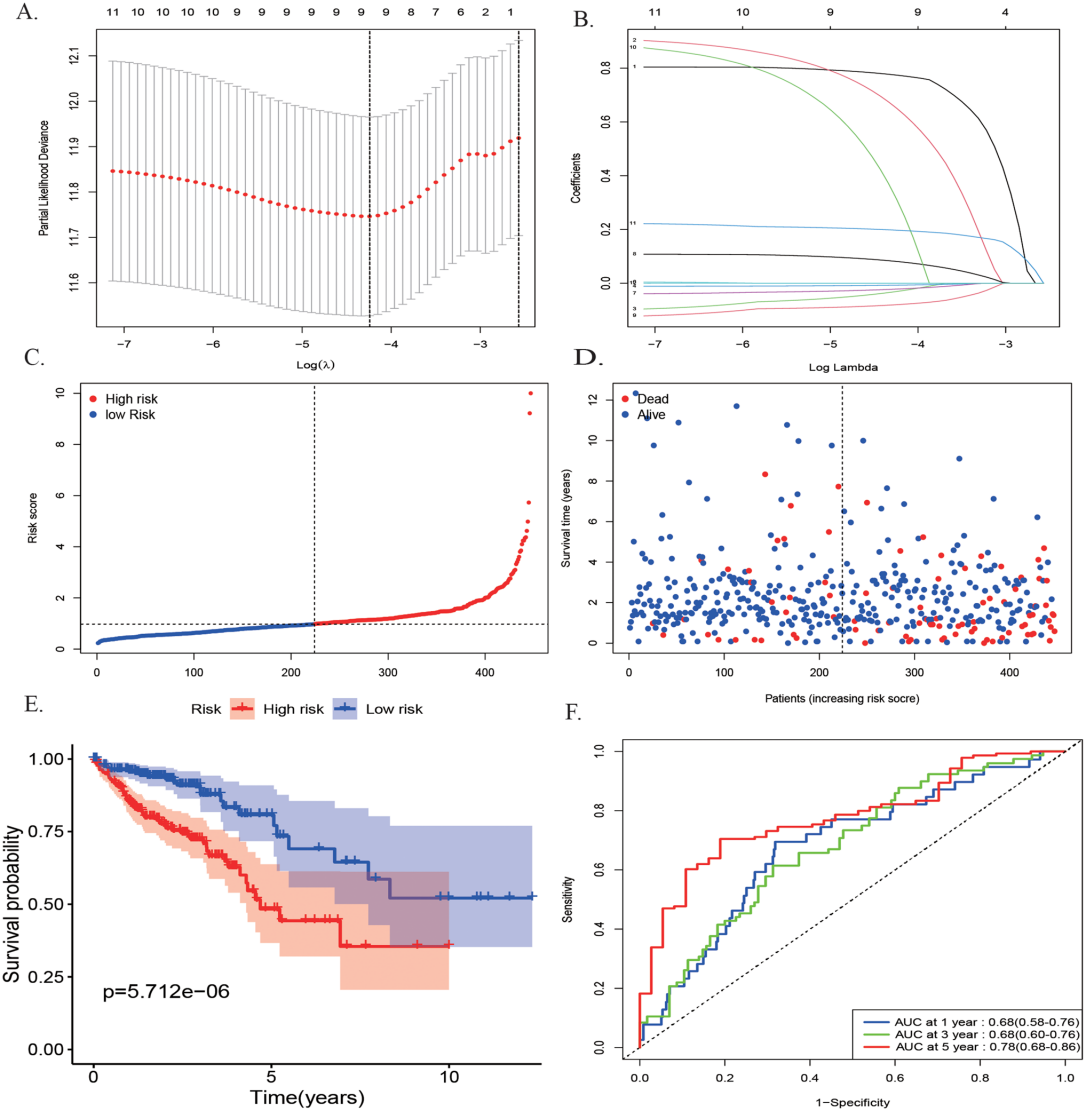
Construction of a prognostic model in TCGA by LASSO Cox regression analysis. **(A,B)** Selection of the optimal parameter (lambda) in the LASSO model for colorectal cancer. **(C)** The distribution of risk score and patient’s survival time. The black dotted line is the optimum cutoff dividing patients into low-risk and high-risk groups. The red curve represents high risk and the blue curve represents low risk. **(D)** The distribution of risk score and patient’s survival status. **(E)** The high-risk score was related to poorer OS. F ROC analysis of the sensitivity and specificity of the OS.

A nine DE-DRGs signature was constructed through the LASSO Cox regression to minimize overfitting and further narrowed the 12 candidate genes (Fig. 3A,B). Four of these nine DRGs were protective (*ESCO2*, *AXIN2*, *PLK1*, *CDC25C*, Coef < 0), and five were related to high-risk (*IGF1*, *TREX2*, *ALKBH2*, *ESR1*, *MC1R*, Coef > 0) (Fig. 2C). The entire names, locations, main pathways, and related coefficients of these genes are listed in Table S4. The risk scores for each patient were defined as the linear combination of the expression levels of the nine DE-DRGs weighted by their related coefficient derived from multivariate Cox regression, as follows: risk score = (0.7945*IGF1) + (0.8003*TREX2) + (− 0.0575 *ESCO2) + (− 0.0095*AXIN2) + (− 0.0299* PLK1) + (0.0990*ALKBH2) + (− 0.0908 *CDC25C) + (0.6670*ESR1) + (0.2066*MC1R). These patients were then divided into high- (n = 223) and low-risk (n = 224) groups based on the median risk scores. Figure 3C, D presents the relationship between the distribution of the rank of risk scores and the patients’ survival status in the training set, which suggested a higher mortality with increasing risk scores. The Kaplan–Meier survival analysis revealed that these patients with high-risk scores correlated with poorer survival rates than those in the low-risk group (Fig. 3E, P < 0.001). The predictive signatures of areas under the curve (AUCs) of the time-independent ROC curve were 0.68 (95% CI: 0.58–0.76) for 1-year, 0.68 (95% CI: 0.60–0.76) 3-year, and 0.78 (95% CI: 0.68–0.86) for 5-year survival, respectively (Fig. 3F).

### Independent prognostic analysis of 9 DE-DRGs signature in TCGA

**Figure 4:**
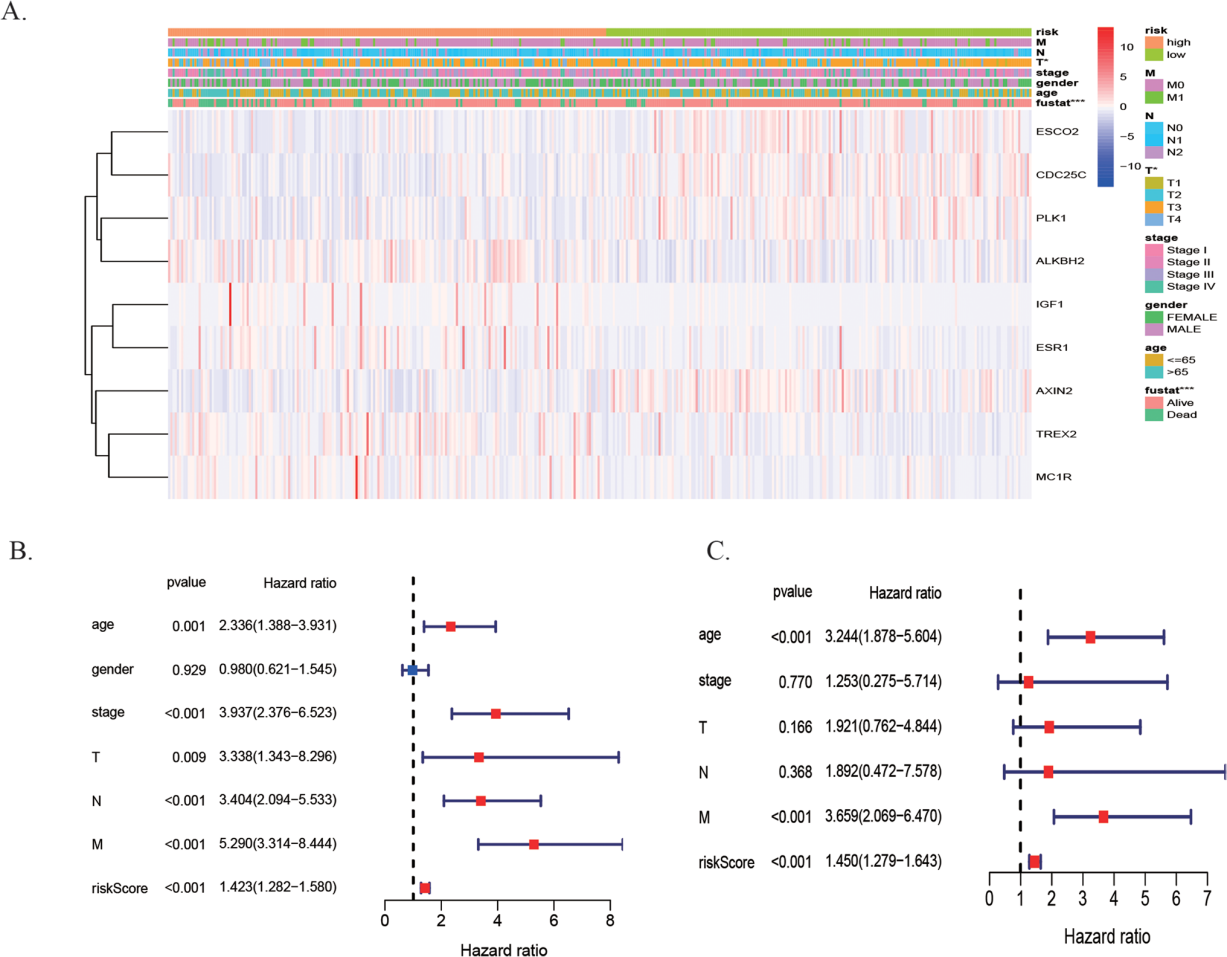
Independent prognostic analysis of 9 DE-DRG signature in the TCGA cohort. **(A)** Heatmap of the DE-DRGs in prognostic signature for TCGA. **(B,C)** Forest plot of the association between risk factors and survival of TCGA-CRC by univariate and multivariate Cox regression analysis.

To test whether the nine DE-DRGs model could be an independent predictive indicator of CRC, 396 patients with detailed clinical features including age, sex, and clinical stage were further analyzed. The expression patterns of nine DE-DRGs in the high- and low-risk groups are displayed in the heatmap depicted in Fig. 4A. The signature was significantly correlated with the cancer invasive depth (T) and survival status. These genes (*ESCO2, AXIN2, PLK1, and CDC25C*) were preferentially expressed in the low-risk group, whereas genes (*IGF1, TREX2, ALKBH2, ESR1,* and *MC1R*) were expressed in the high-risk group. The correlation between age, TNM stage, AJCC stage, and the risk score was statistically significant in the training set through the univariate Cox regression analysis (Fig. 4B). The multivariate Cox regression analysis revealed that the nine DE-DRGs model was identified as an independent prognostic factor (HR = 1.450, 95% CI: 1.279 − 1.642, *P* < 0.001) (Fig. 4C, Table 1). We then classified the patients into subgroups according to age (≤ 65 vs. > 65 years), gender (Female vs. male), T stage (T1–T2 vs. T3–T4), N stage (N0 vs. N1–N2), M stage (M0 vs. M1) and AJCC stage (stage I–II vs. stage III–IV). The stratified results suggested that the high-risk scores closely correlated with poorer survival of patients with CRC, except for T1–T2(Fig. S3A–3L).

**Table 1:** The prognostic value of different clinical characters in the TCGA and GEO.

Variables	Univariate analysis			Multivariate analysis		
	HR	95%CI	P-value	HR	95%CI	P-value
**Training set (TCGA, N = 396)**						
Age (≤ 65 years vs > 65 years)	2.34	1.39–3.93	0.0014	3.24	1.88–5.60	< 0.001
Gender (female vs male)	0.98	0.62–1.54	0.9292	NA	NA	NA
Stage (I–II vs III–IV)	3.94	2.38–6.52	< 0.001	1.25	0.27–5.71	0.7705
T (T1–T2 vs T3–T4)	3.34	1.35–8.30	0.0095	1.92	0.76–4.84	0.1665
N (N0 vs N1–N2)	3.40	2.09–5.53	< 0.001	1.89	0.47–7.58	0.368
M (M0 vs M1)	5.29	3.31–8.44	< 0.001	3.66	2.07–6.47	< 0.001
Risk score (high vs low)	1.42	1.28–1.58	< 0.001	1.45	1.28–1.64	< 0.001
**Validation set (GEO, N = 338)**						
Age (≤ 65 years vs > 65 years)	1.6	1.03–2.49	0.0359	1.37	0.88–2.16	0.163
Gender (female vs male)	0.82	0.53–1.26	0.355	NA	NA	NA
Stage (I–II vs III–IV)	1.44	0.92–2.24	0.1103	NA	NA	NA
T (T1–T2 vs T3–T4)	0.89	0.33–2.44	0.8245	NA	NA	NA
N (N0 vs N1–N2)	1.38	0.89–2.14	0.1551	NA	NA	NA
M (M0 vs M1)	1.78	0.56–5.65	0.3266	NA	NA	NA
Risk score (high vs low)	2.05	1.54–2.73	< 0.001	1.97	1.48–2.64	< 0.001

*NA* not available.

### External validation of 9 DE-DRGs signature in the GEO

Two GEO datasets were merged into a test group, and then we applied the same analysis methods to validate the stability of the predictive model. We found that the number of deaths increased with the increase in risk scores (Fig. S4A,B). Consistently, the survival rate of high-risk patients was poorer than that of low-risk patients (Fig. S4C). The AUCs of 1-,3-, and 5-year were 0.74 (95% CI: 0.60–0.88), 0.68 (95% CI: 0.59–0.76), and 0.72 (95%CI: 0.66–0.79), respectively (Fig. S4D). The heatmap of the nine DE-DRGs in the different risk groups is shown in Fig. S4E. The univariate and multivariate Cox analysis results revealed that the signature was related to OS in the GEO datasets (HR = 2.049, 95% CI: 1.537 − 2.731, *P* < 0.001; HR = 1.974, 95% CI: 1.475 − 2.641, *P* < 0.001, respectively) (Fig. S4F,G, Table 1).

### Analysis of genetic alterations and function enrichment in risk model

**Figure 5:**
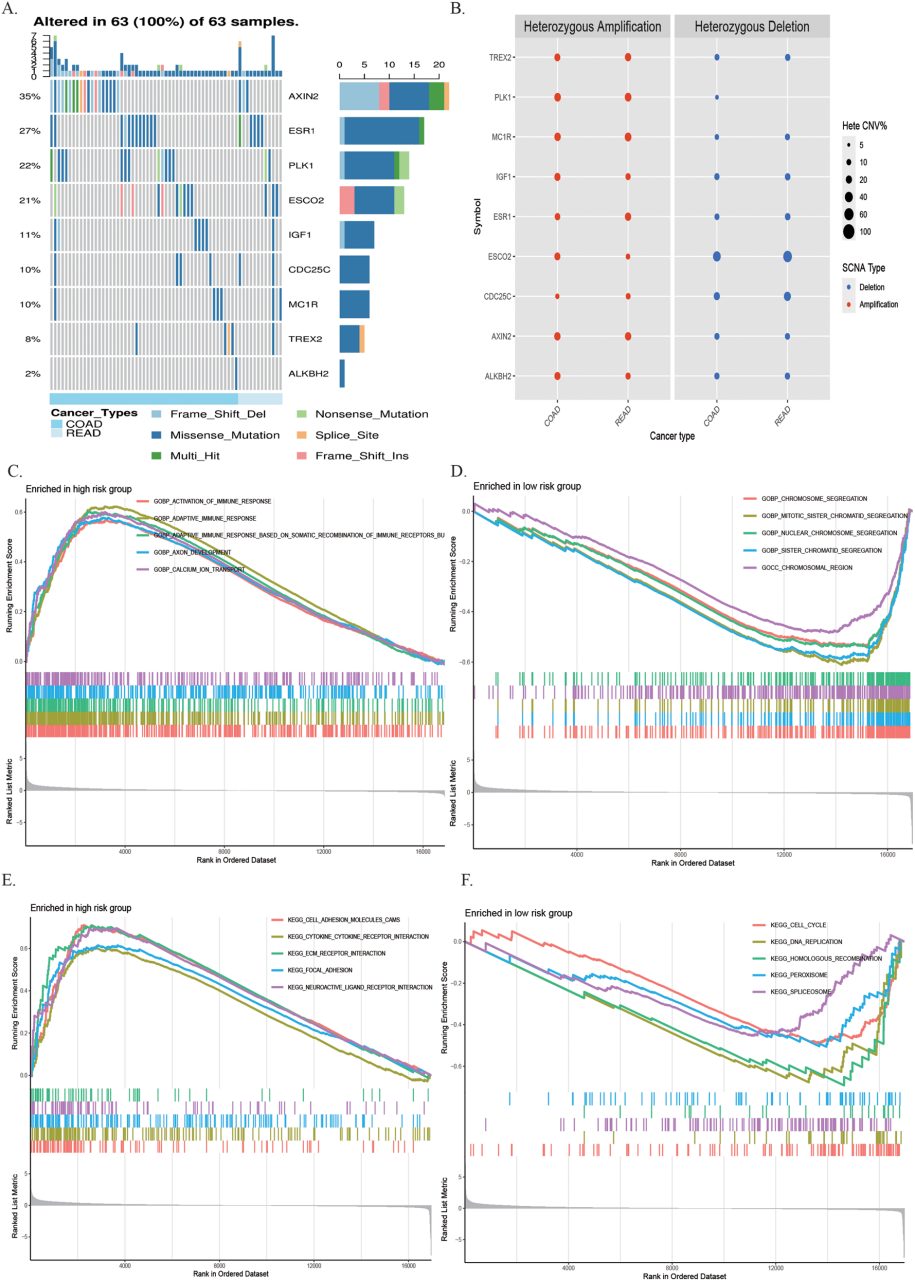
Analysis of genetic alterations, involved signaling pathways and immune correlation of DRGs in CRC. **(A)** Genetic alterations of the 9 DE-DRGs in the CRC cohort. X axis represents cancer type, sky blue indicates COAD, light blue indicates READ. The left Y axis represents ratio of gene mutation, right Y axis represents gene names. Dark blue, cyan, and pink small rectangles indicate the type of gene mutation. **(B–D)** GO and KEGG enrichment analysis of the 9 DE-DRGs. **(D,E)** The scores of 16 immune cells and 13 immune-related functions are displayed in boxplots. CCR, cytokine-cytokine receptor. Adjusted P values were showed as: *ns* not significant; *P < 0.05; **P < 0.01; ***P < 0.001.

The nine DE-DRGs in CRC were altered in 63 of 63 patients (100%), and the major alteration was missense mutation (Fig. 5A), and the CNV results showed the heterozygous CNV frequency among these genes (Fig. 5B) were 5–20% based on GSCA. The frequency of copy number deletion of *ESCO2* was highest in patients with CRC. Function enrichment analysis was applied to clarify the latent biological mechanisms and pathways involved in the risk score signature. The GSEA indicated that the activation of immune responses and adaptive immune response were the main processes in the high-risk group, and chromosome segregation related processes was focused on the low-risk group (Fig. 5C,D). The nine DE-DRGs were mainly enriched in these pathways, including cell adhesion and cytokine-cytokine receptor interaction in high-risk group, and DNA replication and cell cycle were in low-risk group (Fig. 5E,F).

### Correlation between TME and risk model

**Figure 6:**
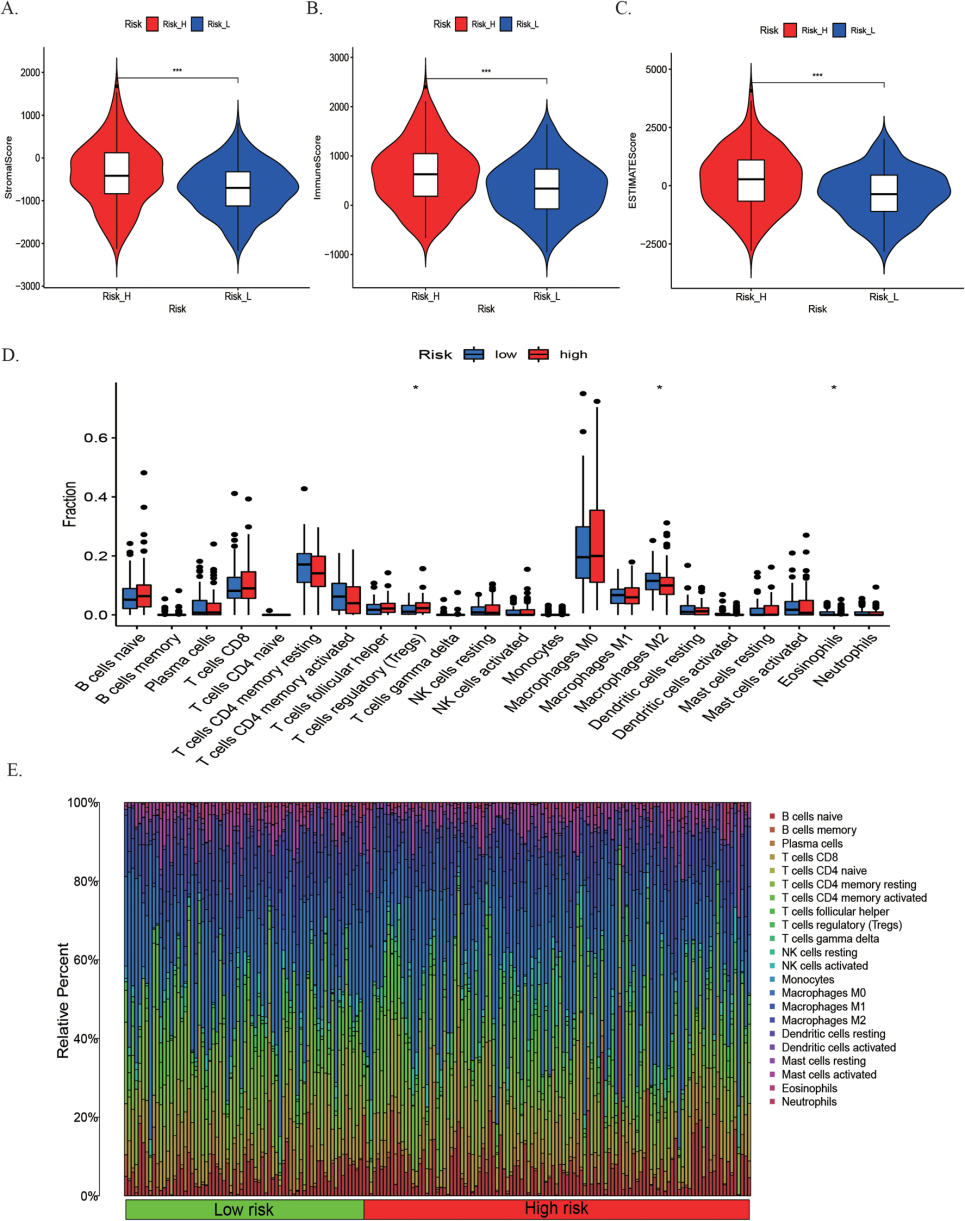
Estimation of the correlation between risk score with TME. **(A–C)** Comparison the stromal score, immune score and ESTIMATE score between high-risk and low-risk groups. **(D)** The scores of 22 immune cells in high-risk and low-risk groups.

We found that the immune score, stromal score, and ESTIMATE score were higher in high-risk group (Fig. 6A–C, *P* < 0.001). The differences of 22 immune cell infiltration levels between the high- and low-risk groups was investigated (Fig. 6D, P < 0.05). The high-risk group had a significant higher abundance of T cells regulatory (Tregs). Macrophages M2 and eosinophils were higher infiltration in the low-risk group. The distribution percentage of 22 immune cells in each patient with CRC was displayed in Fig. 6E.

### Correlation between GDSC drug sensitivity and risk model

**Figure 7:**
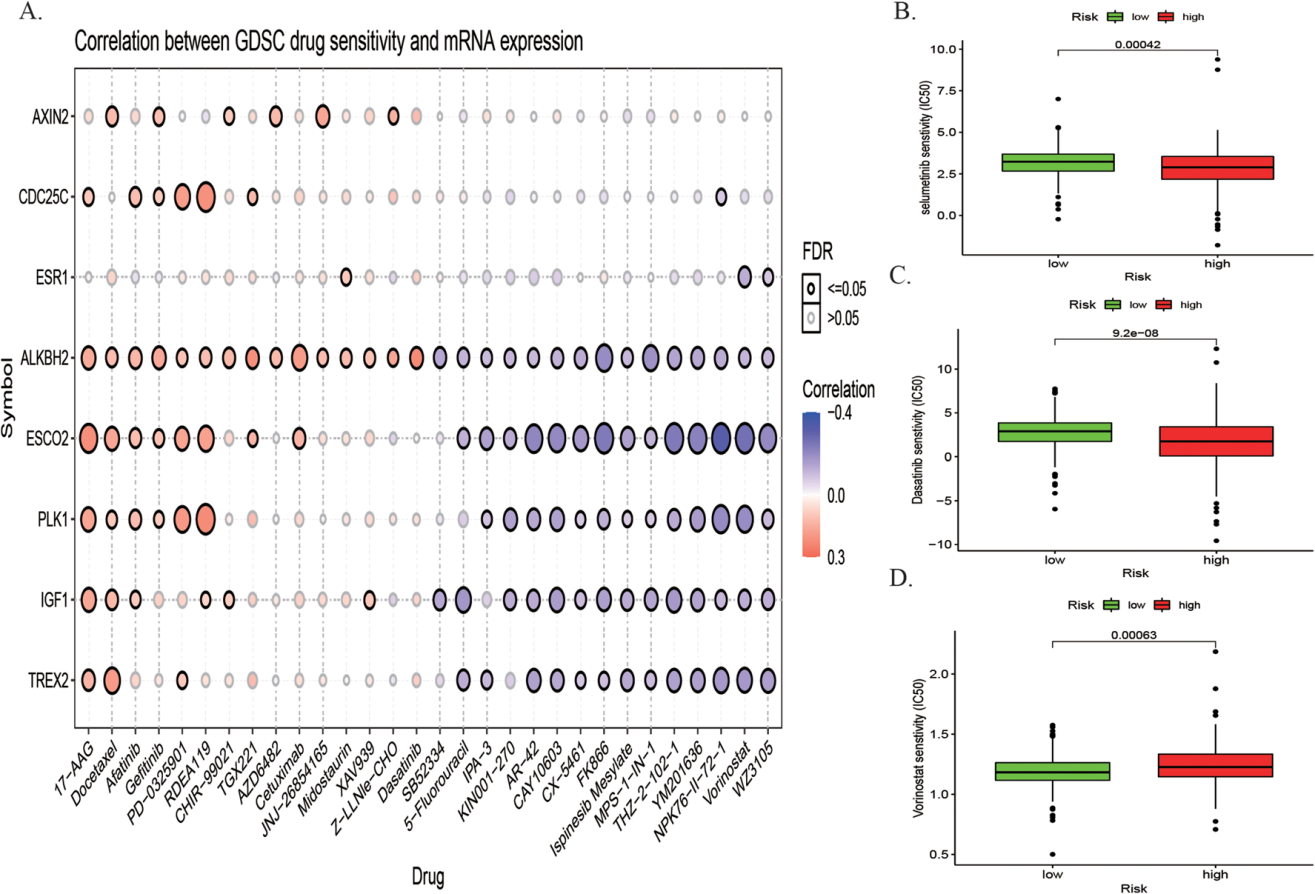
Analysis of drug sensitivity in risk model. **(A)** The correlation between GDSC drug sensitivity and 9 DE-DRGs mRNA expression. **(B–D)** The drug sensitivity of selumetinib, Dasatinib and Vorinostat in high-risk and low-risk groups.

We explored the drug sensitivity response of patients with CRC to chemotherapy and target therapy based on the nine DE-DRGs. These results suggested that there is correlation between the expression of ALKBH2, ESCO2, and PLK1and multiple drugs sensitivity (Fig. 7A). Furthermore, we observed that three target therapy drugs selumetinib, dasatinib and vorinostat witnessed significant differences in estimated IC50 between high- and low-risk groups (Fig. 7B–D). It suggested that low-risk patients were more sensitive to selumetinib and dasatinib but opposite results in vorinostat.

## Discussion

DDR is an endogenous protective mechanism of the human body, which is crucial for maintaining intracellular homeostasis in the face of DNA damage. Dysfunctional DDR may result in genome instability and high gene mutational burden^14^. In recent years, more research has focused on DNA damage, DDR, and CRC tumorigenesis^15–19^. Previous data have shown that exposure of human cell DNA to intrinsic and extrinsic insults, such as oxidative stress, radiation, or chemical exposure, can directly or indirectly impact genomic instability^20,21^. Existing evidence indicates that the accumulation of wrongly repaired or unrepaired DNA damage influences the extracellular and intracellular environment, resulting in inflammation, genetic mutation, cell senescence, death, or even tumorigenesis^22–24^. Some new advances have been made in the studies that explored the relationship between DRGs and cancers. A previous study suggested that gene alterations in DNA repair pathways promote cancer aggressiveness and induce resistance to DNA damage cancer treatments^25^. DNA repair pathways are defenders of the genome, and the involved DRGs exhibit therapeutic capacity and play a vital role in the prognosis of hematologic malignant tumors^26^. Some studies have identified and validated some DRGs to predict the outcome of patients with breast, ovarian, and clear cell renal carcinoma, and found robust predictive ability in these carcinomas^10,27,28^. Hence, DRGs have the potential to be molecular biomarkers for predicting the survival outcomes and improving the diagnosis and treatment of patients with CRC.

Wang et al. have collected DNA repair genes from GSEA to build and validate a 12 gene signature (CCNB3, ISY1, CDC25C, SMC1B, MC1R, LSP1P4, RIN4, TPM1, ELL3, POLG, CD36, and NEK4) based on the expression profiles of TCGA-COAD and GEO datasets (GSE17538 and GSE38832). The authors mainly focused on the relationship between DRGs and colon cancer patients through multivariate cox regression^29^. Compared to this study, we attended to contrast a DRGs signature to assess the OS of patients with CRC. A novel prognostic model based on nine DE-DRGs was firstly identified and validated in TCGA and GEO database through LASSO Cox regression model, and we found the prognostic model was an independent predictive factor for OS in CRC. Our results demonstrated that the prognostic model displayed a more accurate predictive value than previous study. The risk model was closely significant with clinicopathological features and these results indicated that the signature took an advantage in predicting the survival of advanced patients with CRC. It was worth noting that nine genes were included in the TCGA and GEO database. Hence, the model had an ideal prediction impact in the training and test sets. In our study, we also referred to analyses the infiltration of tumor immune cell in different risk groups. Among these genes, *PLK1, CDC25C, ESCO2, AXIN2, TREX2, ALKBH2,* and *MC1R* were upregulated in tumor tissues, whereas *IGF1* and *ESR1* presented downregulation (Figs. S5 and S6). Previous studies based on these genes have been conducted at the molecular level. The role of *PLK1* in carcinogenesis and tumor inhibition remains still controversial^30^. Experimental research has demonstrated that *PLK1* acts as a tumor inhibitor when integrated with certain oncogenes (APC^min)^ in CRC cells, and patients with low *PLK1* expression have a poor prognosis^31^. *CDC25C* participates in regulating cell cycle checkpoint G2/M transition and DNA damage repair^32^. A previous study explored the correlation between CRC and *CDC25C*, which indicated that targeted CDC25C could induce ARID1A-deficient CRC^33^. Studies have reported *ESCO2* can downregulate MMP2 expression to inhibit CRC cell migration and tumor metastasis by mediating the epithelial–mesenchymal transition process^34^. Another study suggested that the transactivation of *AXIN2* inhibited colon cancer cell proliferation and tumor formation by inhibiting Wnt/β-catenin signaling^35^. *IGF1* overexpression can induce lymphangiogenesis and facilitate lymphatic metastasis in CRC cells^36^. Silencing *ESR1* enhanced the chemosensitivity of CRC cell lines to 5-FU^37^. There are no studies on the mechanistic relationship between *TREX2, ALKBH2, MC1R,* and *CRC. TREX2*, a specific 3′-DNA exonuclease expressed in keratinocytes, plays a crucial role in promoting DNA damage repair, inducing cell apoptosis, arousing anti-cancer immunity, and suppressing skin carcinogenesis^38^. A study focused on the link between low DNA methylation of *TREX2* and enhancement of gene expression and shorter survival in laryngeal cancer^39^. *ALKBH2* overexpression inhibits gastric cancer cell proliferation and induces apoptosis and cell cycle arrest^40^. Moreover, *ALKBH2* is associated with chemotherapy and molecular targeting. High expression promotes resistance to temozolomide chemotherapy in glioblastoma cells^41^. These data suggests that *MC1R* high expression is mediated by MITF, which is related to the RAS/ERK-signaling pathway, promoting melanocyte cell division, and enhancing the migration ability of melanoma cells^42,43^. However, the related regulatory mechanism between the three genes mentioned above and CRC occurrence and development need to be elucidated by further research.

Genetic alteration analysis based on GSCA reveal that these genes had very high levels of single nucleotide variants, and missense mutations ranked first. *AXIN2, ESR1, PLK1,* and *ESCO2* change ratios were more than 20% in 63 patients with CRC. These findings suggest that this may be a potential mechanism for the induction of CRC carcinogenesis. GSEA analysis revealed that the main biological progresses of the signature were associated with immune response. In addition, some signaling pathways involved genetic materials synthesis (DNA replication, cell cycle) were enriched. What’s more, the relationship between risk model and tumor immunity was evaluated to find the potential mechanism. Then we found the significant differences in tumor immune score between high- and low-risk patients. Consequently, the high-risk group presented a higher proportion of Tregs. Previous studies had explicated that T cell was correlated with the metastasis of CRC, and DNA damage can induce type I IFN in CRC^44,45^. With the progress of CRC, the chemotherapeutic effects and common target therapy are extremely limited. New types targeted therapeutic drugs need to develop to alleviate the advanced CRC patients. Therefore, we predict the drug response to targeted therapy in high- and low-risk patients, and found that patients with CRC in high-risk were more sensitive to vorinostat (Histone deacetylase (HDAC) inhibitor, was used in Cutaneous T-cell lymphoma) than low-risk patients^46^. This result means that patients in high-risk group can benefit from the small molecular drugs. These results showed this signature can predict the sensitivety of patients to target therapy but need further investigation.

Compared with previous studies, this is the first study to integrate nine DE-DRGs into a multiple gene signature for predicting the survival of CRC, and to analyze the correlation between immune cell domination, immune-related functions, and CRC prognosis. In addtion, there was an innovation distinguished from previous studies, which we analyzed the correlation between the response of drug therapy and risk model and brought new sights on the therapeutic strategy on CRC patients. However, this study has some limitations. First, this study is a bioinformatics analysis based on public databases (TCGA and GEO) and some retrospective studies biases; therefore, more large-scale multicenter cohorts need to be further explored to validate the model. Second, rigorous basic mechanistic research on DRGs and CRC should be carried out to support the signature.

## Methods and materials

### Data sources

The training set of RNA-sequence datasets and the corresponding clinical characteristics of patients with CRC were acquired from TCGA database (https://portal.gdc.cancer.gov/)^47^. The RNA-seq data from TCGA contained 488 patients with CRC and 42 normal colorectal tissues. The mRNA expression data of GSE87211 (n = 363) and GSE103479 (n = 156) were obtained from the GEO database as validation groups (https://www.ncbi.nlm.nih.gov/geo/)^48^. In this study, a total of 513 DRGs were retrieved from a previous study^10^ and the UALCAN database, and are presented in Table S1. All data annotation and extraction were performed using the R software (version 4.0.2). The Perl program and “sva” package were applied to merge two GEO microarray datasets.

Furthermore, this study was also approved by the institutional review committee of the First Affiliated Hospital of Xi’an Jiaotong University, Shaanxi Province, Xi’an, China. Informed consent was renounced because the study did not involve specimen collection and the patients involved in the public databases have obtained ethical approval. And the study was conducted in accordance with the relevant guidelines and the regulations.

### Construction and validation of a prognostic-associated DNA repair-relative gene signature

The differentially expressed genes (DEGs) associated with DNA repair in CRC were identified with the “limma” R package. The DE-DRGs were screened using the criteria: false-positive discovery (FDR) < 0.05, and Log2 | (fold change, FC) |> 1. The univariate Cox regression analysis was used to estimate survival-associated DRGs using the “survival” package (*P* < 0.05). The protein–protein interaction network was created using the online database “STRING” (version 11.0), and the interaction score was set as 0.40, and the cytoHubba application from Cytoscape software was utilized to identify the hub genes^49^. The LASSO Cox regression analysis was performed to construct a predictive signature^50^. The R package "glmnet" was utilized to achieve the variable selection and shrinkage of the LASSO algorithm^51^. The risk scores of each patient were established using the following formula: Risk score = sum (coefficients* expression of gene n)^52^. The "survminer" and "survival" R packages were applied to conduct the Kaplan–Meier survival curve. A time-dependent ROC curve was implemented to assess the predictive ability of the prognostic model by using the "survival ROC" R package^53^. The independent predictive efficiency of the prognostic signature was evaluated using univariate and multivariate Cox analyses. Differences were considered statistically significant at a bilateral *P* < 0.05. The hazard ratios (HRs) and 95% confidence intervals (CIs) were calculated. The merged GEO datasets with survival information were used for external validation. The same methods were used to estimate the risk scores for each case.

### Genetic alterations and function enrichment analysis

The role of genetic alterations in this predictive model was explored through gene set cancer analysis (GSCA) Lite (http://bioinfo.life.hust.edu.cn/web/GSCALite/)^54^. To find relative biological functions and potential molecular pathways regulated by the DE-DRGs signature between the different risk groups, GSEA analysis was performed using "clusterprofler" R package^55,56^. The statistical significance was set at *P* < 0.05. ESTIMATE was performed to calculate the stromal score and immune scores, which illustrated immune cell infiltration in tumor^57^. In addition, CIBERSORT algorithm and “limma” package were used to analyze the differences between the different risk groups and 22 types tumor-infiltrating immune cells.

### Chemotherapy response and small molecular drug prediction

The drug sensitivity response to chemotherapy and target therapy in patients with CRC were determined based on public database GDSC (Genomics Drug sensitivity in cancer)^58^. The half-maximal inhibitory concentration (IC50) was evaluated to represent the drug response. The GSCA was used to investigate the underlying drugs based on nine DE-DRGs. In addition, the package “pRRophetic” was applied to estimate the potential target drugs between the high- and low-risk groups.

### Statistics analyses

All statistical analyses were performed using the R software (version 4.0.2). Continuous variables were expressed as mean ± SE, whereas categorical variables were summarized as frequency (n) and proportion (%). The Wilcoxon signed-rank test was used to compare the DRGs signature mRNA expression levels between cancer and non-cancer samples. Bilateral *P* value < 0.05 was considered statistically significant.

## Conclusion

In conclusion, we developed a novel, valid and reliable prognostic model based on nine DE-DRGs. The signature was significantly correlated with OS in the training and validation cohorts, which provided a novel perspective for the diagnosis and treatment of CRC.

## Supplementary Information

Supplementary Figures.

Supplementary Tables.

## Data Availability

The datasets generated and analyzed during the current study are available from public databases, TCGA (http://cancergenome.nih.gov/abouttcga), and GEO (https://www.ncbi.nlm.nih.gov/geo/) databases.

## Abbreviations

AUC: The area under curve
CRC: Colorectal cancer
CI: Confidence interval
DEGs: Differentially expressed genes
DE DRGs: Differentially expressed DNA repair genes
GEO: Gene Expression Omnibus
GDSC: Genomics drug sensitivity in cancer
GSCA: Gene set cancer analysis
GSEA: Gene set enrichment analysis
HR: Hazard ratio
LASSO: Least absolute shrinkage and selection operator
MITF: Melanogenesis associated transcription factor
OS: Overall survival
TCGA: The Cancer Genome Atlas
