# Supplementary material for: Identification and validation of prognosis-associated DNA repair gene signatures in colorectal cancer
Source: Sci Rep. 2022 Apr 28;12:6946. doi: 10.1038/s41598-022-10561-w (PMC9050689; doi:10.1038/s41598-022-10561-w)
Supplement: Supplementary file 1 — Supplementary Figures. [file 41598_2022_10561_MOESM1_ESM.pdf]

Figure Legends

Figure S1. The heatmap of 118 DE-DRGs in TCGA.

Figure S2. Forest plot of 36 survival-related DRGs from TCGA-CRC by univariate Cox regression analysis.

Figure S3. Stratification analysis of various clinical features by Kaplan-Meier survival curves for the patients with CRC in the TCGA dataset. A-B. age; C-D. gender; E-F. T stage; G-H. N stage; I-J.M stage; K-L. AJCC stage.

Figure S4. External validation of the 9 DE-DRGs signature in the GEO datasets. A. The distribution of risk score and patient's survival time. B. The distribution of risk score and patient's survival status. C. The high-risk score was related to poorer OS. D. ROC analysis of the sensitivity and specificity of the OS. E. Heatmap of the DNA repair-related gene expression profiles in prognostic signature for TCGA. F-G. Forest plot of the association between risk factors and survival of TCGA-CRC by univariate and multivariate Cox regression analysis.

Figure S5. Gene expression between Tumor and Normal tissues in TCGA-CRC cohort. ***P<0.001

Figure S6. The protein expression of 7 DRGs between colorectal normal and tumor tissues of in HPA (ESCO2 and MC1R cannot be found in HPA).

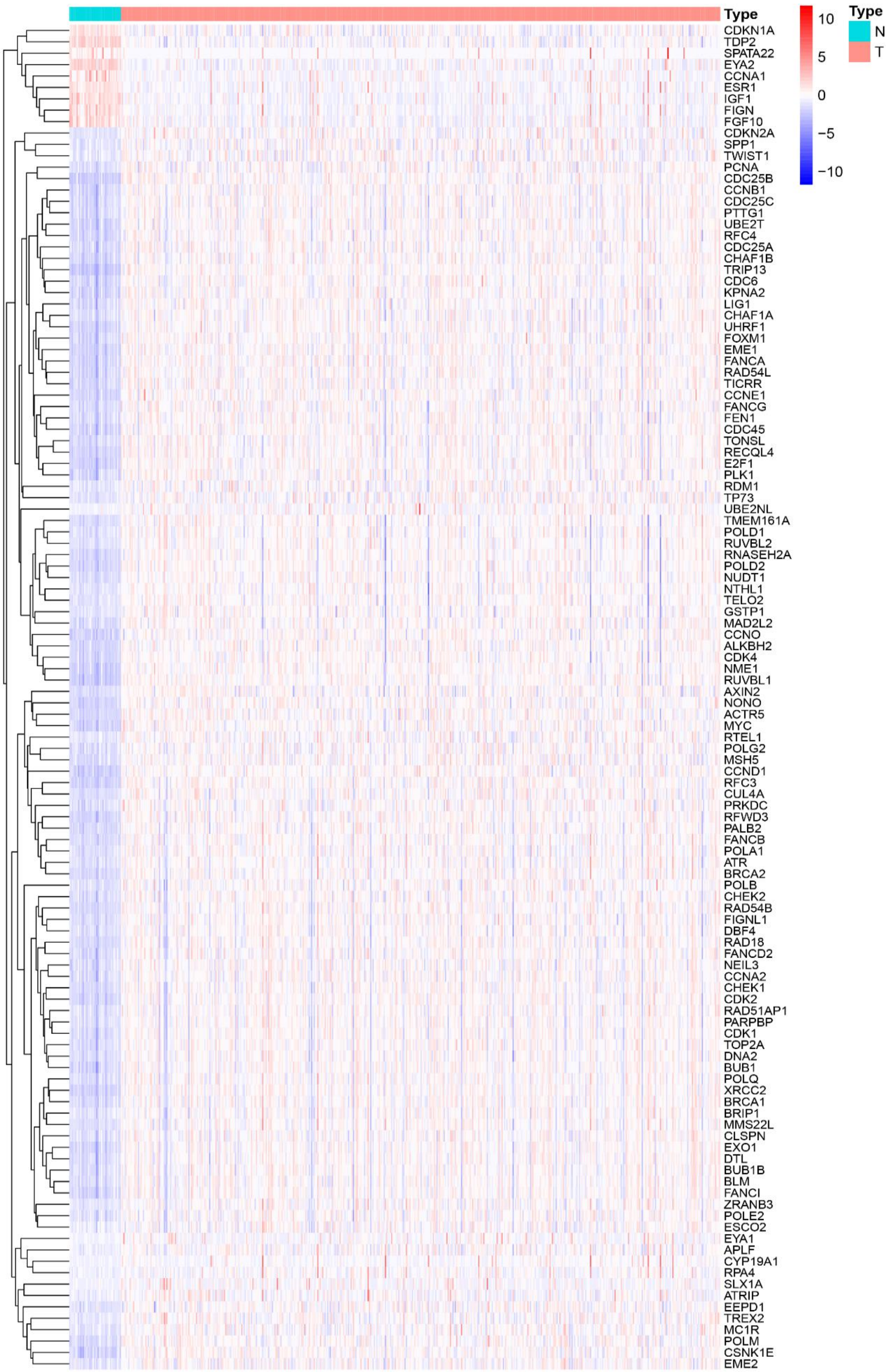

Figure S1. The heatmap of 118 DE-DRGs in TCGA.

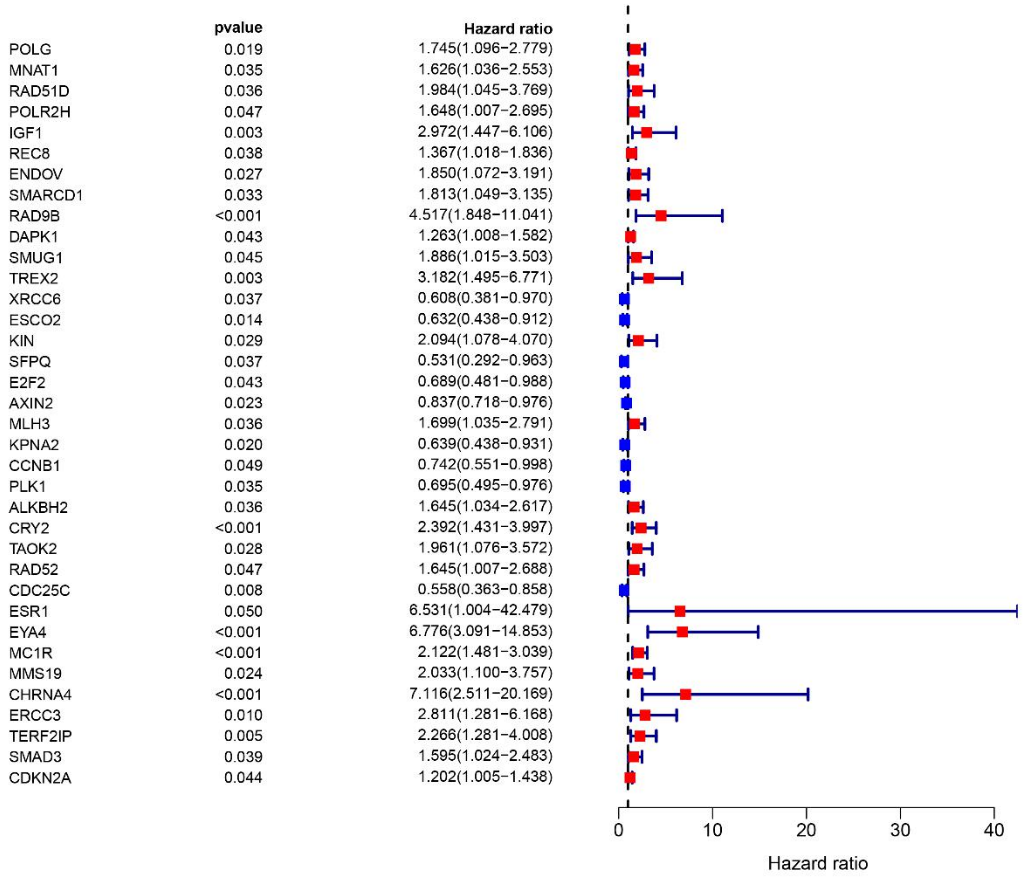

Figure S2. Forest plot of 36 survival-related DRGs from TCGA-CRC by univariate Cox regression analysis.

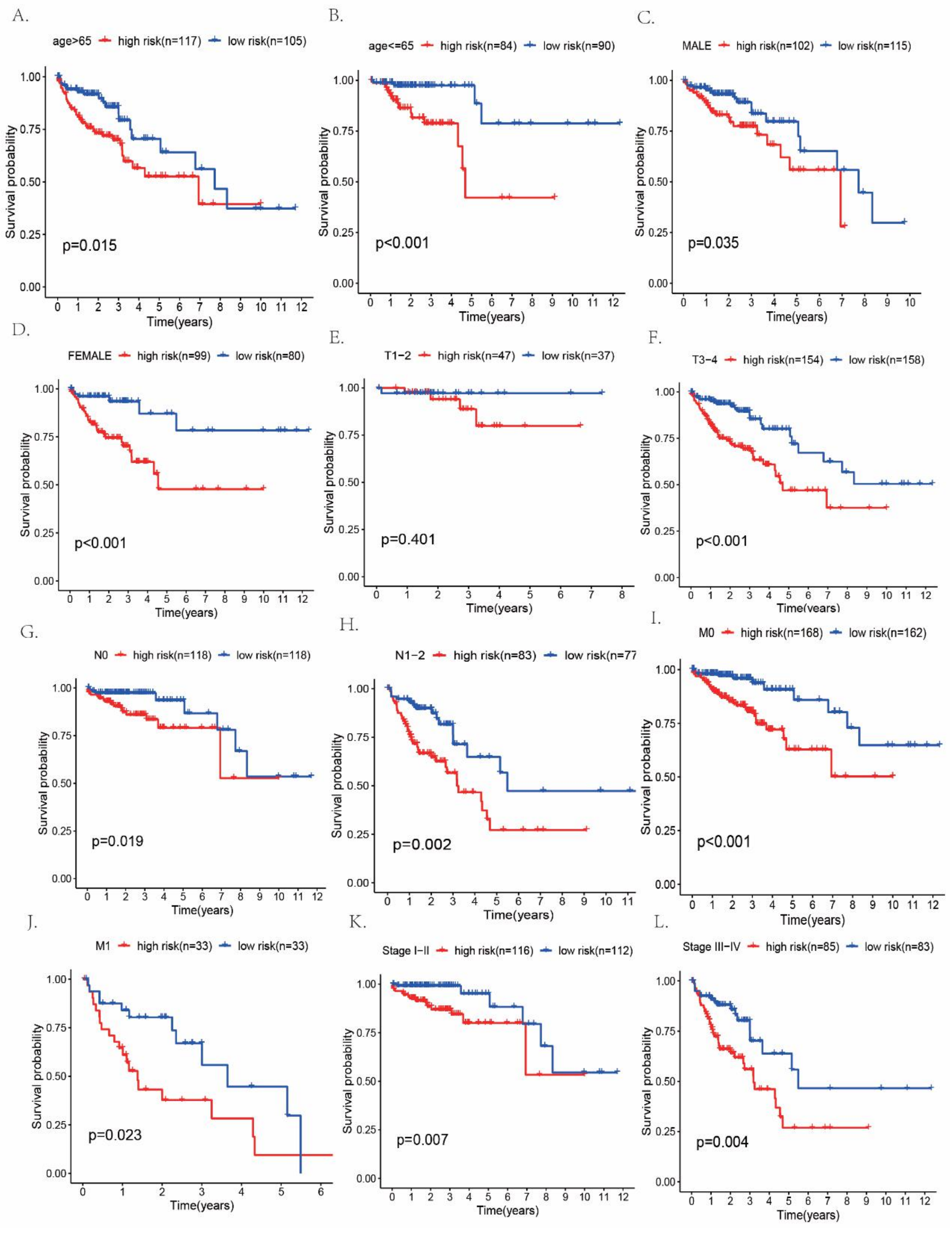

Figure S3. Stratification analysis of various clinical features by Kaplan-Meier survival curves for the patients with CRC in the TCGA dataset. A-B. age; C-D. gender; E-F. T stage; G-H. N stage; I-J.M stage; K-L. AJCC stage.

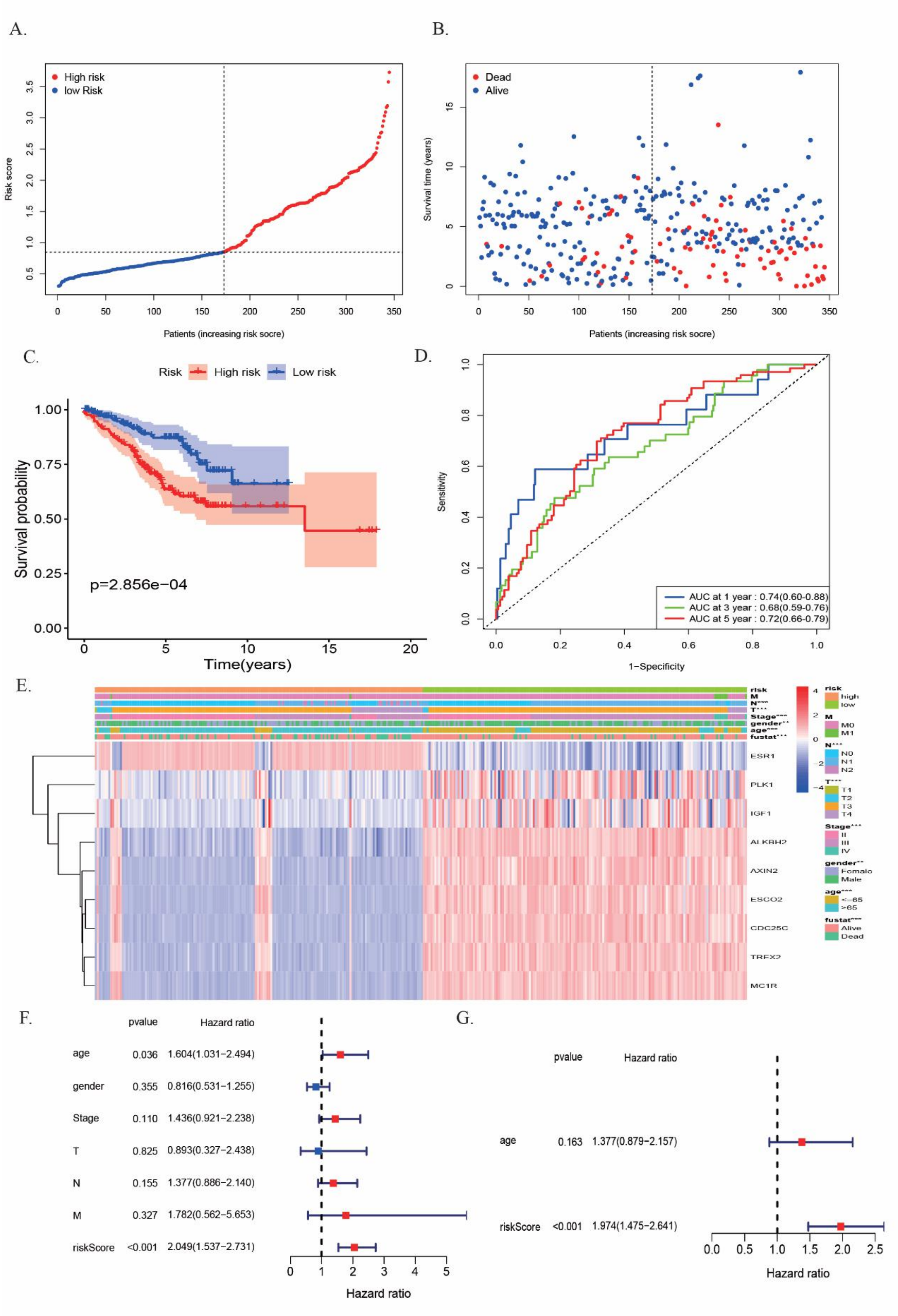

Figure S4. External validation of the 9-DRGs signature in the GEO datasets. A. The distribution of risk score and patient's survival time. B. The distribution of risk

score and patient's survival status. C. The high-risk score was related to poorer OS. D. ROC analysis of the sensitivity and specificity of the OS. E. Heatmap of the DNA repair-related gene expression profiles in prognostic signature for TCGA. F-G. Forest plot of the association between risk factors and survival of TCGA-CRC by univariate and multivariate Cox regression analysis.

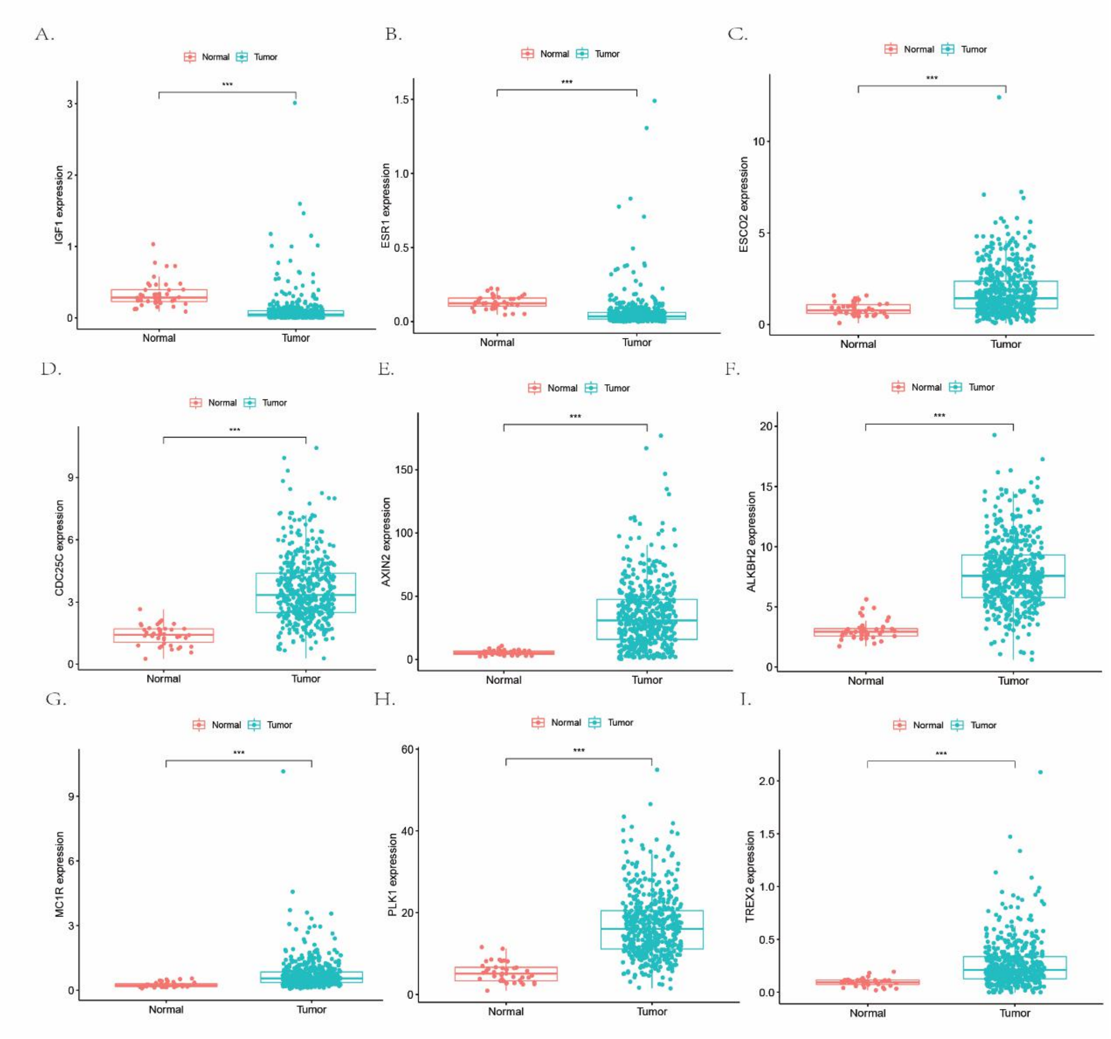

Figure S5. Gene expression between Tumor and Normal tissues in TCGA-CRC cohort. ***P<0.001

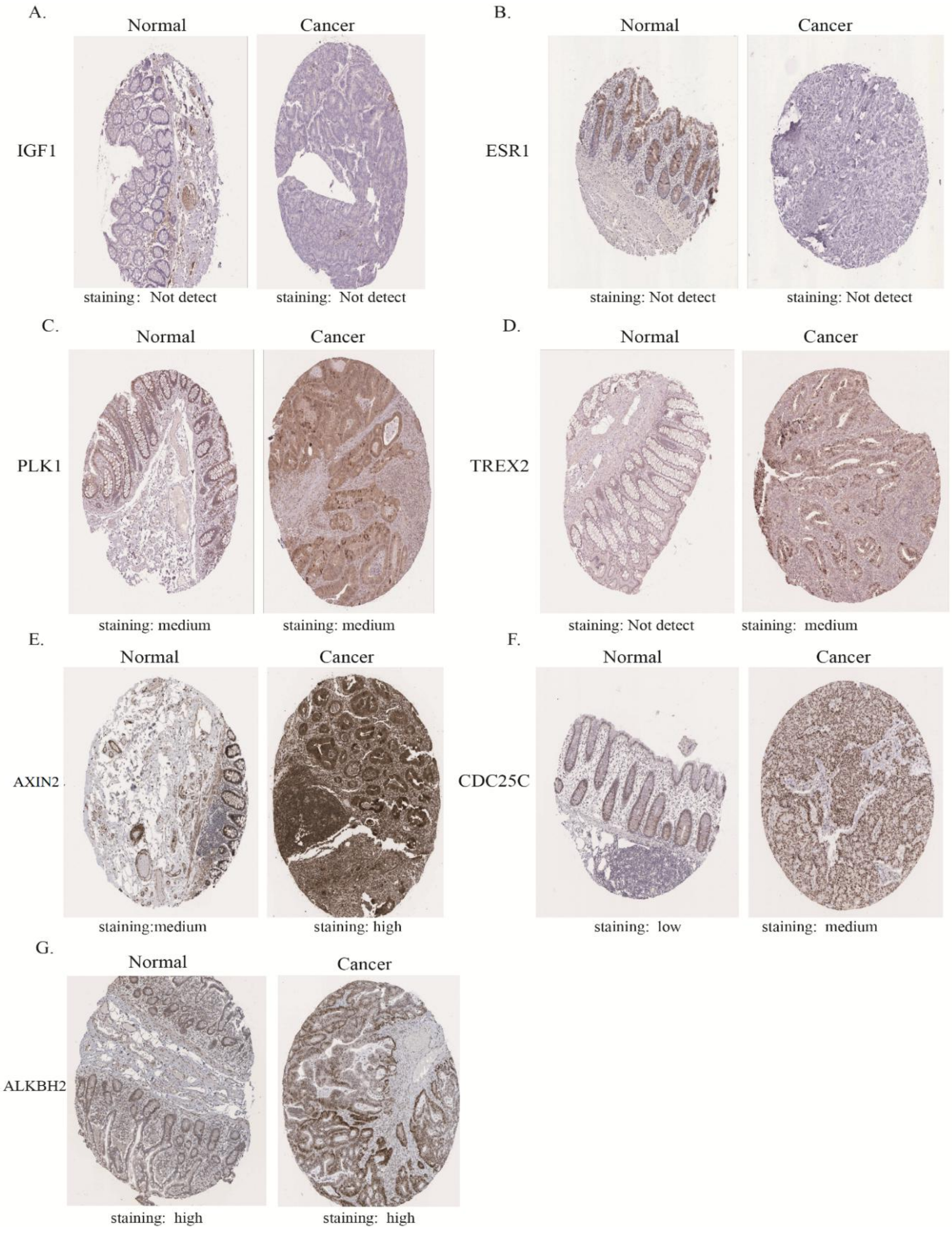

Figure S6. The protein expression of 7 DRGs between colorectal normal and tumor tissues of in HPA (ESCO2 and MC1R cannot be found in HPA).
