# Supplementary material for: Identification and validation of prognosis-associated DNA repair gene signatures in colorectal cancer
Source: Sci Rep. 2022 Apr 28;12:6946. doi: 10.1038/s41598-022-10561-w (PMC9050689; doi:10.1038/s41598-022-10561-w)
Supplement: Supplementary file 2 — Supplementary Tables. [file 41598_2022_10561_MOESM2_ESM.pdf]

Table S1. Baseline characteristics of patients with CRC in the TCGA and GEO.

Characteristics		TCGA(n=396)	GEO(n=338)
Survival time(month)		28.30±1.25*	59.05±2.03*
Age (years)	≤65	174(43.9%)	162(47.9%)
	＞65	222(56.1%)	176(52.1%)
Gender	Male	217(54.8%)	142(42.0%)
	Female	179(45.2%)	196(58.0%)
T stage	T1	12(3.0%)	1(0.3%)
	T2	72(18.2%)	11(3.3%)
	T3	273(68.9%)	279(82.5%)
	T4	39(9.9%)	47(13.9%)
N stage	N0	236(59.6%)	144(42.6%)
	N1-3	160(40.4%)	195(57.4%)
M stage	M0	330(83.3%)	328(97.0%)
	M1	66(16.7%)	10(3%)
Stage	I	74(18.7%)	0
	II	154(38.9%)	141(41.7%)
	III	102(25.7%)	187(55.3%)
	IV	66(16.7%)	10(3%)
Survival status	Alive	321(81.1%)	253(74.9%)
	Dead	75(18.9%)	85(25.1%)

*The data are presented as mean ± SE.

Table S2. The list of 493 DNA repair-related genes.

Gene Names					
SOD1	POLR2D	ZFYVE26	FZR1	SSRP1	ESR1
ASF1A	SMARCA4	KIAA1429	XRCC4	ZRANB3	DCLRE1A
POLG	HUWE1	SWSAP1	FOS	RDM1	UHRF1
FBXO6	DMC1	CDKN1A	UBE2N	CRY1	FANCL
CSNK1E	XRCC2	RNF169	SETMAR	UBE4B	SMAD4
EP300	CHEK1	POLR2J	MLH1	TTC5	EYA4
CDH13	FANCB	DUSP3	SMAD2	MGME1	PTTG1
MNAT1	PARG	CCNE1	FAN1	CDC25A	UBA52
RAD51AP1	POLD1	HMGB1	RNF168	RAD9A	RFC4
EEPD1	CCNA1	HINFP	NEIL1	SETD2	IKBKG
BAP1	MSH5	INO80D	ATXN3	FANCD2	MC1R
MEN1	POLR2A	PARPBP	POLD3	SMARCB1	TYMS
RAD51B	POLR2B	TRIP13	POLH	PARP1	GTF2H2C
ZBTB32	PSMD3	POLE2	MORF4L2	CASP3	MMS19
RPAIN	FIGNL1	POLM	PPP2R5A	APEX2	SHPRH
RAD51D	NFKB1	CHD4	POLD4	FEN1	PALB2
SPATA22	SPO11	RPA3	E2F2	POLN	GTF2H4
DDB1	CEP170	PPP4C	HERC2	EME1	GADD45A
NTHL1	ATRX	PRMT6	TREX1	SMC4	KAT5
HDAC1	CDK7	REV3L	UBE2V2	RAD23A	GPS1
PRKDC	SMARCD1	MPG	AXIN2	WWP1	EXO5
SMURF2	RBBP8	BARD1	ERCC2	PPP2R5C	BCCIP
NABP2	KIF22	POLR2G	UBE2B	ALKBH2	TMEM161A
DDR1	XAB2	STAT1	WDR33	NSMCE2	AP5Z1
HUS1	SUMO1	TP53BP1	TAOK1	GTF2H2	POLA1
E2F4	LIG1	ESCO2	POLB	SMC1A	UVSSA
SP1	RPS27L	TEX15	USP47	JUN	XRCC3
FANCA	UBE2D3	UBB	XRCC5	SFR1	CCNA2
UBE2I	HUS1B	XPC	GSTP1	INO80	CDK2
RFWD3	LIG4	FGF10	MEIOB	CDKN1B	RBX1
POLR2H	TRIP12	PPP1CA	DDB2	CDK1	TCEA1
CHEK2	MSH2	ASTE1	MDC1	NEIL2	COPS5
POLQ	TOP1	PNKP	POLR2K	USP7	PARP4
SMARCA2	RB1	RHNO1	RNF8	MTA1	CCNH
POLG2	YY1	RELA	CINP	PPP2R5E	SMC5
SPP1	AKT1	PPM1D	RUVBL1	FANCG	RAD21
TELO2	HELQ	RFC1	USP1	ERCC6L2	FTO
ATR	ASCC3	TDG	MLH3	MAD2L2	EPC2
SMC3	MSH4	BLM	CDC14B	WRN	JMY
POLR2E	RFC3	RFC5	DNA2	SMC2	ATF2
RAD54L	TWIST1	KIN	CCNO	MDM4	NUDT1
GEN1	UIMC1	SMARCC2	POLR2C	BRAP	POLR2F

GTF2H5	EXO1	FHIT	SMARCA1	UVRAG	CHRNA4
TP53	POLR2I	RAD18	SYCP1	BUB1	POLL
USP28	DOT1L	MUTYH	BRCA1	CRY2	BABAM1
ERCC8	PMS2	RBBP4	MMS22L	WDR48	PMS1
SLC30A9	FANCM	DTX3L	PPP2R5D	TICRR	MED17
FIGN	CDC6	DYRK2	GTF2H3	MCPH1	RPS3
IGF1	RAD9B	XPA	MGMT	SPRTN	DBF4
SWI5	MUS81	WWP2	RAD54B	TAOK2	CDC25B
TAOK3	CHD1L	CUL4B	KPNA2	TNP1	NINL
REC8	UPF1	PCNA	E2F1	CEP164	DEK
TOP2A	DHX9	PARP9	DCLRE1C	LIG3	MYC
UNG	ERBB2	SIRT6	CCNB1	SMARCAD1	EGFR
RAD51	ATM	PLK3	CRB2	ALKBH1	PPP2R5B
ERCC6	SMARCA5	INIP	RPS27A	RAD52	EYA3
INTS3	ERCC4	BRIP1	SMC6	CSNK1D	RBBP7
RTEL1	NONO	SFPQ	KDM2A	EME2	RASSF1
APTX	FANCF	CHAF1B	ZNF350	FANCI	RECQL4
SLX4	ABL1	ERCC5	CHAF1A	CDC25C	CUL4A
PRPF19	CYP1A1	ATMIN	PARP3	CLSPN	PARP2
XRCC1	POLE	USP3	ALKBH3	UBE2NL	ERCC3
UBE2T	ATRIP	UBE2U	NHEJ1	RNASEH2A	RPA1
MCM9	CDKN2D	INO80E	POLK	POLD2	GTF2H1
WRNIP1	BAZ1B	BAX	MSH3	SPIDR	EYA2
SUPT16H	HIC1	AATF	BRCA2	NEK11	UBC
POLE3	WEE1	CCND1	TERF2	TDP1	RECQL5
RAD17	RAD51C	POLI	RPA2	CDC45	RRM2B
NCOA6	UBA1	FOXM1	TEX12	CREBBP	HDAC2
RAD50	TOP3A	DMAP1	NEIL3	ZSWIM7	SETX
RFC2	EYA1	RUVBL2	MDM2	E2F6	AP5S1
FANCC	DAPK1	NEK1	RAD23B	DTL	TERF2IP
NSMCE1	TONSL	SMG1	TDP2	POLE4	NBN
NME1	UBE2A	DCLRE1B	MBD4	TP73	SMAD7
RBM14	SMUG1	SMARCD2	CEBPG	ACTR5	GADD45G
IFI16	ETS1	APEX1	RPA4	BUB1B	APLF
RECQL	FANCE	VCP	TOPBP1	OGG1	CIB1
SLX1A	TREX2	HMGB2	OTUB1	POLDIP3	REV1
PPP2R2A	XRCC6	BRCC3	MORF4L1	RAD1	NABP1
ENDOV	CYP19A1	CETN2	PLK1	SIRT1	ERCC1
PPP4R2	TERF1	SMAD3	CREB1	BTG2	DDX1
CDK4	CDKN2A	POLR2L	IRS1	ESCO1	IGHMBP2
MSH6					

Table S3. The list of 118 differentially expressed DNA repair-related genes.

Gene Names					
CSNK1E	GSTP1	TONSL	TP73	NME1	FEN1
RAD51AP1	RUVBL1	TREX2	ACTR5	SLX1A	EME1
EEPD1	DNA2	CYP19A1	BUB1B	XRCC2	ALKBH2
SPATA22	CCNO	CDKN1A	ESR1	CHEK1	CDK1
NTHL1	BRCA1	CCNE1	UHRF1	FANCB	FANCG
PRKDC	MMS22L	PARPBP	PTTG1	POLD1	MAD2L2
FANCA	RAD54B	TRIP13	RFC4	CCNA1	BUB1
RFWD3	KPNA2	POLE2	MC1R	MSH5	TICRR
CHEK2	E2F1	POLM	PALB2	FIGNL1	EME2
POLQ	CCNB1	ESCO2	TMEM161A	EYA1	DTL
POLG2	CHAF1A	FGF10	POLA1	POLB	APLF
SPP1	BRCA2	BLM	CCNA2	CDKN2A	CDK4
TELO2	NEIL3	RAD18	CDK2	LIG1	FANCI
ATR	TDP2	PCNA	NUDT1	RFC3	CDC25C
RAD54L	RPA4	BRIP1	DBF4	TWIST1	CLSPN
FIGN	PLK1	CHAF1B	CDC25B	EXO1	UBE2NL
IGF1	ZRANB3	CCND1	MYC	CDC6	RNASEH2A
TOP2A	RDM1	FOXM1	RECQL4	NONO	POLD2
RTEL1	CDC25A	RUVBL2	CUL4A	ATRIP	CDC45
UBE2T	FANCD2	AXIN2	EYA2		

Table S4. Features of genes in the prognostic gene signature.

Gene symbol	Full name	Location	Expression	Risk coefficent
IGF1	Insulin like growth factor 1	Chromosome 12	Down	0.7945
TREX2	Three prime repair exonuclease 2	Chromosome X	Up	0.8003
ESCO2	Establishment of cohesion 1 homolog 2	Chromosome 8	Up	-0.0575
AXIN2	Axin protein 2	Chromosome 17	Up	-0.0095
PLK1	Polo like kinase 1	Chromosome 16	Up	-0.0299
ALKBH2	Alkb, alkylation repair homolog 2	Chromosome 12	Up	0.099
CDC25C	Cell division cycle 25 homolog C	Chromosome 5	Up	-0.0907
ESR1	Estrogen receptor 1	Chromosome 6	Down	0.667
MC1R	Melanocortin 1 receptor	Chromosome 16	Up	0.2066
